# Treatment for type 2 diabetes and diabetic nephropathy by targeting Smad3 signaling

**DOI:** 10.7150/ijbs.87820

**Published:** 2024-01-01

**Authors:** Huijun He, Honglian Wang, Xiaocui Chen, Yu Zhong, Xiao Ru Huang, Ronald CW Ma, Cheng Wang, Hui-Yao Lan

**Affiliations:** 1 Division of Nephrology, Department of Medicine, The Fifth Affiliated Hospital of Sun Yat-sen University, Zhuhai, Guangdong, 519000, China; Guangdong Provincial Key Laboratory of Biomedical Imaging, The Fifth Affiliated Hospital of Sun Yat-sen University, Zhuhai, Guangdong Province, 519000, China; 2 Department of Medicine and Therapeutics, and Li Ka Shing Institute of Health Sciences, the Chinese University of Hong Kong, Hong Kong; and Guangdong-Hong Kong Joint Laboratory on Immunological and Genetic Kidney Diseases, and Departments of Nephrology and Pathology, Guangdong Provincial People's Hospital, Guangdong Academy of Medical Sciences, Guangzhou, Guangdong 510080, China.

**Keywords:** Type-2 diabetes, Smad3 inhibitor, Treatment, Islet, Nephropathy

## Abstract

TGF-β/Smad3 signaling plays a critical role in type 2 diabetes (T2D) and type 2 diabetic nephropathy (T2DN), but treatment by specifically targeting Smad3 remains unexplored. To develop a new Smad3-targeted therapy for T2D and T2DN, we treated db/db mice at the pre-diabetic or established diabetic stage with a pharmacological Smad3 inhibitor SIS3. The therapeutic effect and mechanisms of anti-Smad3 treatment on T2D and T2DN were investigated. We found that anti-Smad3 treatment on pre-diabetic db/db mice largely attenuated both T2D and T2DN by markedly reducing blood glucose levels, and inhibiting the elevated serum creatinine, microalbuminuria, and renal fibrosis and inflammation. Unexpectedly, although SIS3 treatment on the established diabetic db/db mice inhibited T2DN but did not significantly improve T2D. Mechanistically, we uncovered that inhibition of T2DN in SIS3-treated db/db mice was associated with effectively restoring the balance of TGF-β/Smad signaling by inhibiting Smad3 while increasing Smad7, thereby suppressing Smad3-mediated renal fibrosis and NF-κB-driven renal inflammation via lncRNA Erbb4-IR and LRN9884-dependent mechanisms. We also revealed that inhibition of islet β cell injury by preventing the loss of islet Pax 6 could be the mechanism through which the pre-diabetic treatment, rather than the late SIS3 treatment on db/db mice significantly improved the T2D phenotype.

## Introduction

Type 2 diabetes (T2D) is a global health problem 1-3. Its onset and progression are multifactorial but largely due to insufficient insulin secretion by pancreatic β-cell dysfunction and insulin resistance in the target organs 3, 4. Diabetic nephropathy (DN) is one of the major microvascular complications of type-2 diabetic nephropathy (T2D) and the leading cause of end-stage kidney disease 5. Despite remarkable advances in our understanding of the pathogenesis of T2D, the incidence of T2D and the prevalence of T2DN are still increasing in the past decades 1, 3, 5. Therefore, it is urgently needed to identify novel pathological mediators and therapeutic targets to prevent or delay the progression of T2D and T2DN.

TGF-β is a master regulator in renal fibrosis and mediates T2DN via its downstream Smad3-dependet mechanism 6, 7. In chronic kidney disease including T2DN, Smad3 is highly activated and correlates with progressive kidney injury 6-9. Thus, systemic blockade of TGF-β signaling with neutralizing anti-TGF-β1 antibodies has been developed for the treatment of both type 1 diabetic nephropathy (T1DN) and T2DN 10-13. However, it is very disappointing that such experimental findings cannot be translated to the clinical use as a recent randomized, double-blind, phase 2 clinical study fails to show the efficacy of the anti-TGF-b antibody treatment on patients with T2DN 14. Results from this clinical study suggest that targeting the upstream TGF-β signaling with anti-TGF-β antibodies may not be a good strategy for treatment of T2DN. Our recent finding that mice overexpressing human latent TGF-β1 are protected from the development of streptozotocin-induced T1DN fully supports this clinical notion 15, and demonstrates a diverse role between latent versus active TGF-β1 in the pathogenesis of diabetic kidney disease 6, 7. This may well explain why the antibody-based anti-TGF-β treatment fails in treating patients with T2DN clinically. In addition, TGF-β/Smad signaling can also be activated in the diabetic kidney by many mediators including angiotensin II, advanced glycation end products, and C-reactive protein via the Smad3 crosstalk pathways 16-18. Moreover, recent evidence also suggests that GSK3β is a key regulator of the TGF-β/Smad signaling activation in progressive chronic kidney disease 19. Thus, treatment for T2DN should target the downstream TGF-β signaling molecules such as Smad3 that directly regulates T2DN, rather than the general effect of TGF-β signaling.

The pathogenic role for Smad3 in T2D and T2DN comes from our recent studies that genetic deletion of Smad3 from db/db mice can protect against the development of T2D and T2DN as well as myocardial disease and diabetic dyslipidemia 20-23. Furthermore, we also find that transplantation with the Smad3 null islets can also largely improve the therapeutic effect on both T1D and T2D and its complications 24. These findings imply that specifically targeting Smad3 may be a good therapeutic strategy for treatment of T2D and T2DN, which was examined in the present study by treating diabetic db/db mice with a Smad3-specific inhibitor SIS3. It has been reported that SIS3 can block TGF-β1-induced fibrotic response by inhibiting Smad3 phosphorylation and DNA binding 25.

Increasing evidence shows that prediabetes is a high-risk factor for the development of T2D and diabetic complications 26. In the United States, about 25% of adolescents and young adults have prediabetes 26. This may be associated with the incidence and prevalence of youth-onset T2D and its complications. Indeed, youth-onset T2D is common in families of racial/ethnic minorities with more rapid and progressive complications 27, 28. Thus, the early preventive treatment in prediabetic adolescents and young adults may be more beneficial in avoiding the progression of T2D and T2DN. In the present study, we not only examined the therapeutic effect of SIS3 on diabetic db/db mice but also treated the young db/db mice at the pre-diabetic stage from the age of 4 weeks when levels of blood glucose remain not significantly elevated without obvious insulin resistance. The therapeutic effect and mechanisms of SIS3 on T2D and T2DN in pre-diabetic versus diabetic db/db mice were investigated and compared.

## Results

### SIS3 treatment on pre-diabetic db/db mice protects against the progression of T2D and T2DN

**Figure 1:**
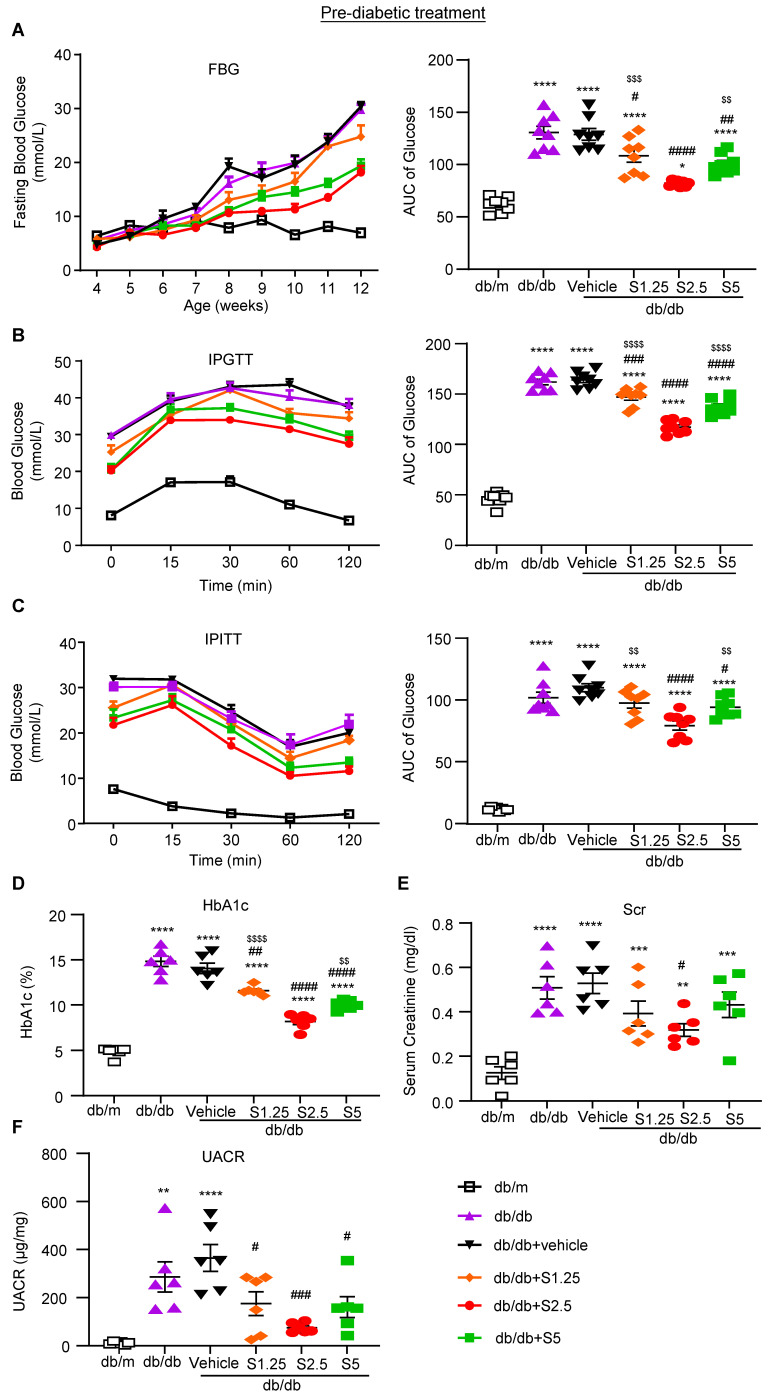
** Prediabetic treatment of db/db mice with SIS3 from the age of 4 weeks to 12 weeks largely improves hyperglycemia, glucose tolerance, insulin resistance and renal functional injury. (A)** Fasting blood glucose and area under curve (AUC). **(B, C)** Blood glucose levels and AUC during intraperitoneal glucose tolerance test (IPGTT) and intraperitoneal insulin tolerance test (IPITT). **(D)** HbA1c levels. **(E)** Serum creatinine. **(F)** Urinary albumin/creatinine ratio (UACR). Data represents the mean ± SEM for at least 6 mice per group.^ *^P<0.05, ^**^P<0.001, ^***^P<0.001, ^****^P<0.0001 versus normal db/m mice; ^#^*P*<0.05, ^##^*P*<0.01, ^###^*P*<0.001, ^####^*P*<0.0001 compared with the control-treated db/db mice (vehicle). ^$$^*P*<0.01, ^$$$^*P*<0.001, ^$$$$^*P*<0.0001 compared with the S2.5-treated db/db mice. SIS3 dosages used: S1.25=SIS3 1.25 mg/kg.bw; S2.5=SIS3 2.5 mg/kg.bw; S5=SIS3 5 mg/kg.bw.

We first examined whether anti-Smad3 treatment can effectively protect against the development of T2D and T2DN by treating the pre-diabetic db/db mice from the age of 4 weeks. We first determined an optimal therapeutic dose of SIS3 on T2D and diabetic nephropathy. Groups of 8 male db/db mice were treated with a daily SIS3 at dosages of 1.25, 2.5 and 5 mg/kg body weight intraperitoneally from the age of 4 weeks to 12 weeks. Interestingly, compared to the control solvent-treated db/db mice (vehicle), prediabetic treatment with SIS3 dose-dependently protected liver from injury by significantly reducing serum levels of AST levels and a trend decrease in ALT without alteration of LDH and body weight (Supplementary Figure S1). Similarly, treatment with SIS3 also dose-dependently reduced the levels of fasting blood glucose (FBG), improved glucose tolerance as determined by the glucose tolerance test (IPGTT) and insulin resistance and insulin sensitivity as measured by the insulin tolerance test (IPITT), with the best dose at 2.5 mg/kg (Figure 1A-C). Of note, the use of higher dose of SIS3 at 5 mg/kg did not produce any more therapeutic benefits in terms of FBG, IPGTT, and IPITT while increasing AST when compared with SIS3 at the dose of 2.5 mg/kg (Figure 1 and Supplementary Figure S1A). As hemoglobin A1C (HbA1c) is a useful index for average blood glucose measurement over the previous 8-12 weeks and is associated with the onset or progression of microvascular complications in patients with T2D 29, 30, we also examined the blood HbA1c levels and found that pre-diabetic treatment with SIS3 dose-dependently reduced the HbA1c level in db/db mice compared with the control vehicle-treated db/db mice, again with the most effective dose at 2.5mg/kg (Figure 1D).

**Figure 2:**
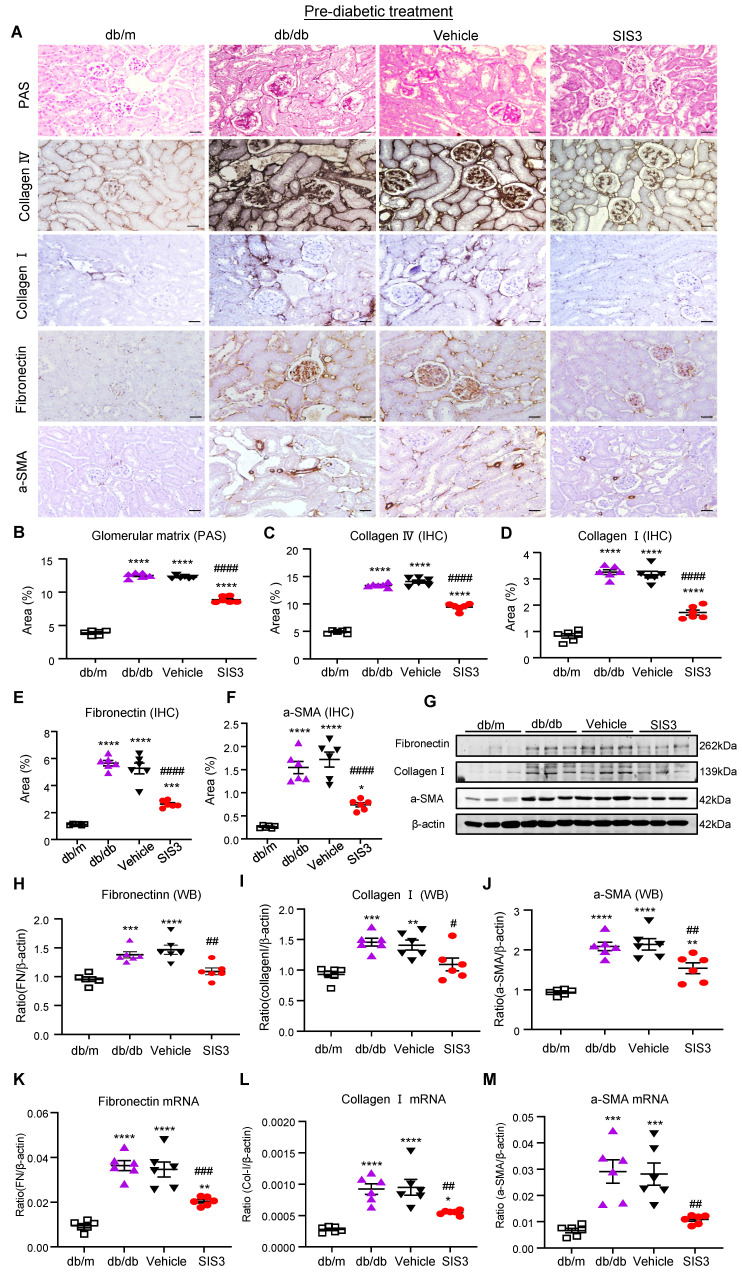
** Prediabetic treatment of db/db mice with SIS3 from the age of 4 weeks to 12 weeks protects against renal fibrosis. (A)** PAS and immunostaining with antibodies against collagens I and IV, fibronectin (FN), and α-SMA in the week 12 kidney of db/m and db/db mice treated with or without SIS3 at a dose of 2.5 mg/kg/day. **(B-F)** Semi-quantitation of glomerular matrix deposition and accumulation of collagens I, IV, fibronectin, and α-SMA+ myofibroblasts from the immunostained sections. **(G-J)** Western blotting and quantitative analysis of renal fibrosis including collagen I, α-SMA, and fibronectin. **(K-M)** Real-time PCR analysis for mRNA expression of fibronectin, collagen I, and α-SMA in the kidney of db/m or db/db mice at 12 weeks. Data represents the mean ± SEM for at least 6 mice per group. ^**^P<0.01, ^***^P<0.001, ^****^P<0.0001 versus normal db/m mice. ^#^*P*<0.05, ^##^*P*<0.01, ^###^*P*<0.001, ^####^*P*<0.0001 compared with the control-treated db/db mice (vehicle). SIS3 dosages used: SIS3 =SIS3 2.5 mg/kg.bw. Scale bar, 50μm.

We next examined the therapeutic effect of SIS3 on the development of T2DN in pre-diabetic db/db mice at the age of 4 weeks and found that SIS3 treatment for 8 weeks (4-12 weeks) could dose-dependently protect against the development of T2DN by lowing the serum levels of creatinine and urine albumin-to-creatinine ratio (UACR), again with the best inhibitory effect on renal dysfunctions at 2.5mg/kg (Figure 1E, F). Thus, the SIS3 dosage at 2.5 mg/kg/day was selected as the best dose for the entire study. As shown in Figure 2, PAS-staining and immunohistochemistry demonstrated that pre-diabetic treatment with SIS3 at the dose of 2.5mg/kg/day protected against the development of T2DN by largely reducing the glomerular matrix expansion and progressive renal fibrosis such as extensive accumulation of collagen I, IV, FN, and α-SMA+ myofibroblasts (Figure 2A-F). These inhibitory effects on renal fibrosis were also further confirmed by western blot and real-time PCR analysis that the SIS3 prediabetic treatment largely suppressed collagen I, FN, and α-SMA mRNA and protein expression (Figure 2G-M).

**Figure 3:**
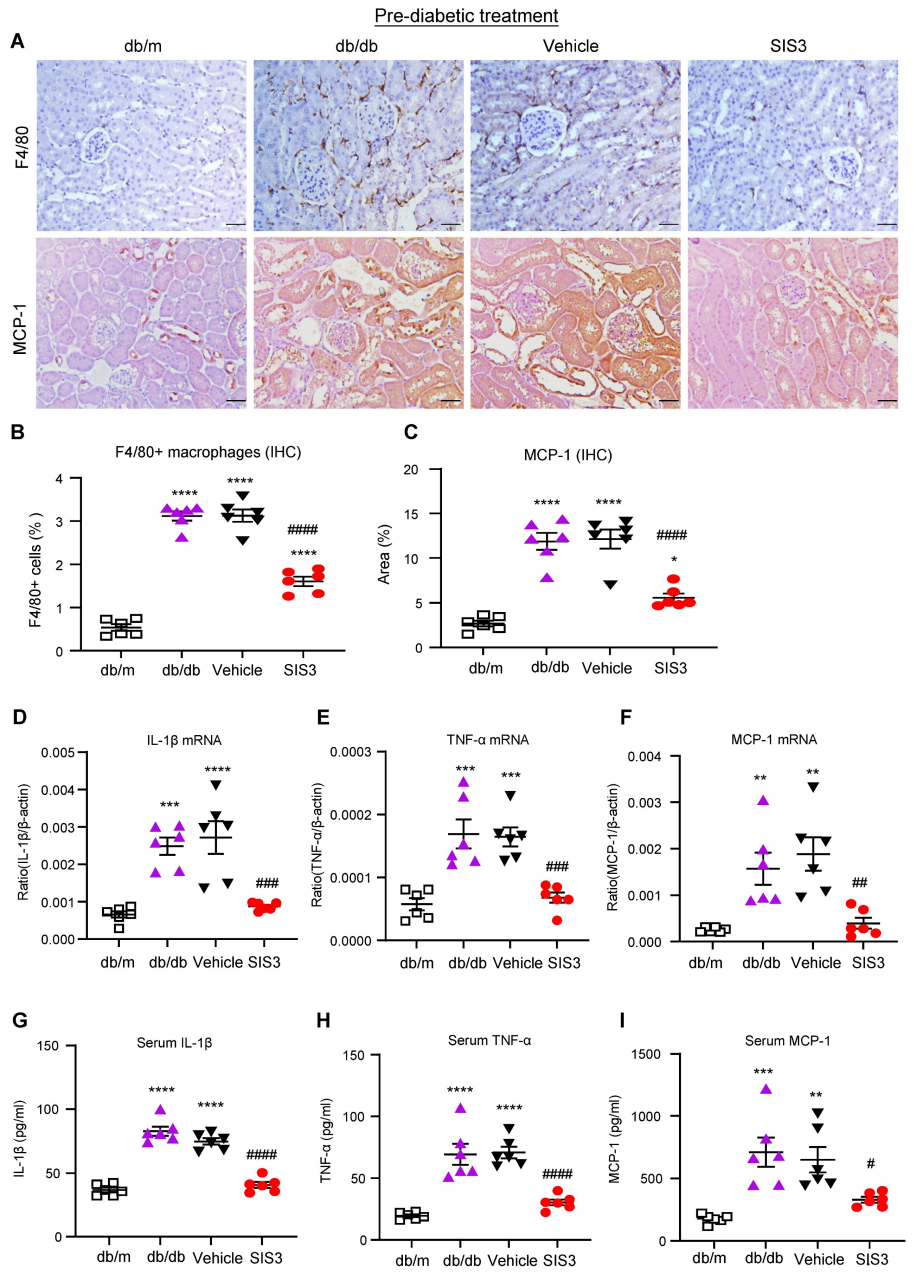
** Prediabetic treatment of db/db mice with SIS3 from the age of 4 weeks to 12 weeks protects against renal inflammation. (A-C)** Immunostaining and quantitative analysis of F4/80+ macrophages and expression of MCP-1 in the kidney of db/m and db/db mice at 12 weeks of age. **(D-F)** Real-time PCR analysis detects IL-1β, TNF-α, and MCP-1 mRNA expression in kidney of db/m and db/db mice at 12 weeks. **(G-I)** ELISA analysis detects serum levels of IL-1β, TNF-α and MCP-1 in db/m and db/db mice at 12 weeks. Data represents the mean ± SEM for at least 6 mice per group. ^*^P<0.05, ^**^P<0.01, ^***^P<0.001, ^****^P<0.0001 versus normal db/m mice. ^#^*P*<0.05, ^##^*P*<0.01, ^###^*P*<0.001, ^####^*P*<0.0001 compared with the control-treated db/db mice (vehicle). SIS3 dosages used: SIS3=SIS3 2.5 mg/kg.bw. Scale bar, 50μm.

It is well known that T2D and diabetic complications are associated with low-grade inflammation. We then examined if SIS3 treatment on prediabetic db/db mice is also associated with inhibition of systemic and renal inflammation. Immunohistochemical staining and real-time PCR detected that db/db mice at 12 weeks developed significant renal inflammation by increasing F4/80+ macrophages and a marked upregulation of MCP-1, IL-1β, and TNF-α, which was largely blocked by prediabetic treatment with SIS3 (Figure 3A-F, Supplementary Figure S6). ELISA analysis also showed that diabetic db/db developed systemic inflammation at the age of 12 weeks as demonstrated by a significant increase in serum levels of IL-1β, TNF-α, and MCP-1, which was also prevented by SIS3 prediabetic treatment (Figure 3 G-I). Taken together, results from this study revealed that SIS3 is an effective therapeutic agent and is capable of targeting Smad3-mediated both T2D and T2DN when it is used earlier in prediabetic db/db mice.

### Treatment with SIS3 partially improves T2D but inhibits T2DN in db/db mice

**Figure 4:**
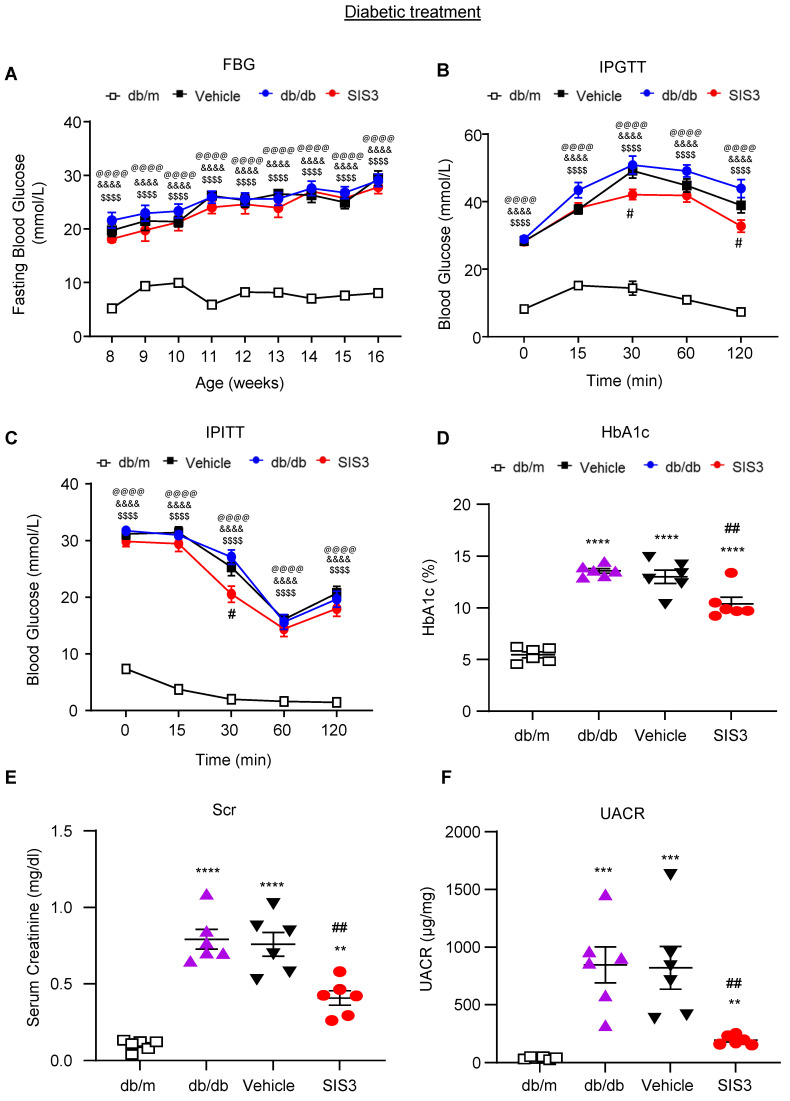
** Treatment with SIS3 in db/db mice from the age of 8 weeks to 16 weeks shows less effective in controlling hyperglycemia with little improvement in glucose and insulin tolerance but effectively inhibits renal functional injury in db/db mice. (A)** Fasting blood glucose. **(B)** Glucose tolerance test (IPGTT). **(C)** Insulin tolerance (IPITT). **(D)** HbA1c levels at 16 weeks. **(E)** Serum creatinine at 16 weeks. **(F)** Urinary albumin/creatinine ratio (UACR) at 16 weeks. Data represents the mean ± SEM for at least 6 mice per group. ^**^P<0.001, ^***^P<0.001, ^****^P<0.0001 versus normal db/m mice. ^#^*P*<0.05, ^##^*P*<0.01 compared with the control-treated db/db mice (vehicle). ^&&&&^*P*<0.0001: untreated db/db mice versus normal db/m mice. ^$$$$^*P*<0.0001: vehicle-treated db/db mice versus normal db/m mice. ^@@@@^*P*<0.0001: SIS3-treated db/db mice versus normal db/m mice. SIS3 dosages used: SIS3 =SIS3 2.5 mg/kg.bw.

We then investigated the therapeutic effect of SIS3 on T2D and T2DN by treating the established db/db mice with daily SIS3 (2.5 mg/kg body weight) intraperitoneally from the age of 8 weeks to 16 weeks. In contrast to the results seen in the prediabetic treatment, treatment with SIS3 in the established db/db mice showed no inhibitory effects on serum levels of AST and ALT without altering the body weight and LDH levels (Supplementary Figure S2). Furthermore, compared with the vehicle-treated db/db mice, treatment with SIS3 did not alter the fasting blood glucose levels with minimal effect on glucose tolerance and insulin resistance (Figure 4A-C). However, treatment with SIS3 did significantly reduce the blood HbA1c levels in db/db mice at 16 weeks (Figure 4D).

**Figure 5:**
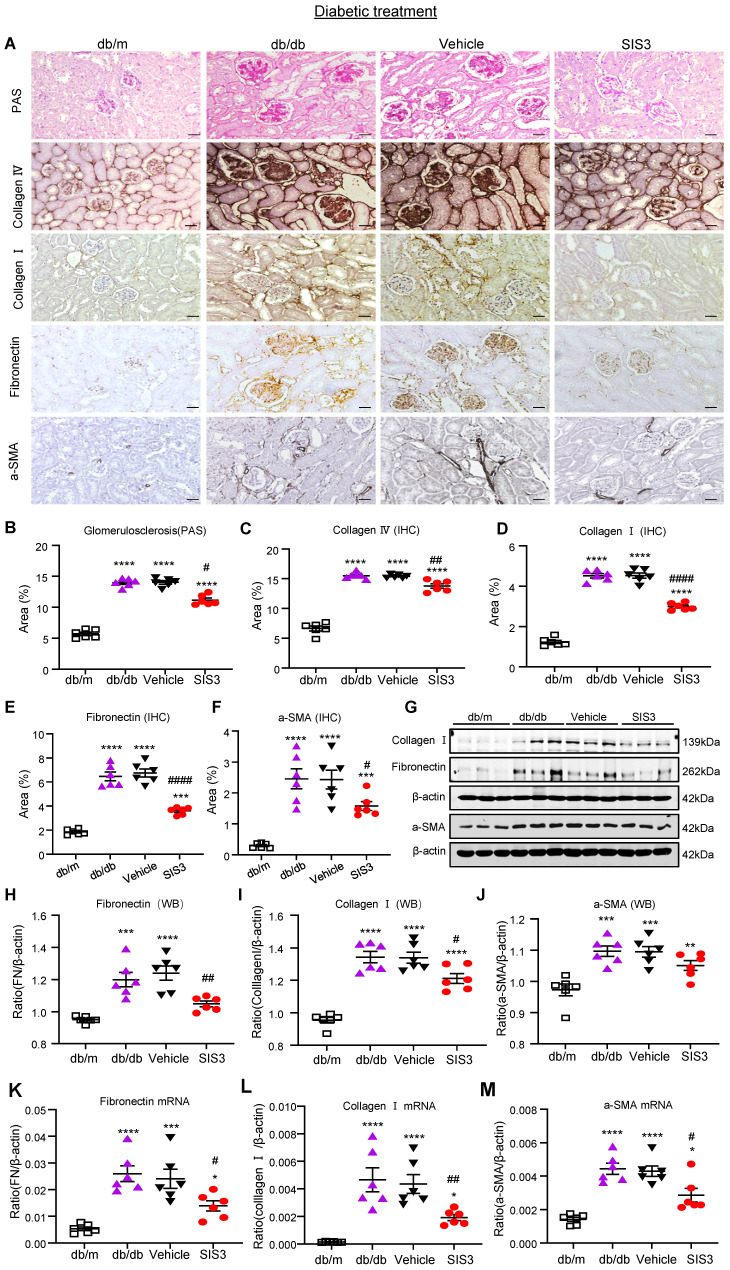
** Treatment with SIS3 in db/db mice from the age of 8 weeks to 16 weeks significantly inhibits renal fibrosis in db/db mice. (A)** PAS and immunostaining with antibodies against collagens I and IV, fibronectin, and α-SMA in the kidney of db/m and db/db mice treated with or without SIS3 at a dose of 2.5 mg/kg/day. **(B-F)** Semi-quantitation of glomerular matrix deposition and accumulation of collagens I, IV, fibronectin, and α-SMA+ myofibroblasts from the immunostained sections. **(G-J)** Western blotting and quantitative analysis of renal fibrosis including collagen I, α-SMA, and fibronectin. **(K-M)** Real-time PCR analysis for mRNA expression of fibronectin, collagen I, and α-SMA in the kidney of db/m or db/db mice at the age of 16 weeks. Data represents the mean ± SEM for at least 6 mice per group. ^*^P<0.05, ^**^P<0.01, ^***^P<0.001, ^****^P<0.0001 versus normal db/m mice. ^#^*P*<0.05, ^##^*P*<0.01, ^####^*P*<0.0001 compared with the control-treated db/db mice (vehicle). SIS3 dosages used: SIS3=SIS3 2.5 mg/kg.bw. Scale bar, 50μm.

We next examined the therapeutic effect of SIS3 on T2DN. Although treatment with SIS3 did not effectively attenuate T2D, unexpectedly, it did produce an inhibitory effect on the development of T2DN by significantly lowering serum levels of creatinine and UACR (Figure 4E, F). Histopathologically, PAS-staining and immunohistochemistry also demonstrated that treatment with SIS3 on the established db/db mice resulted in inhibition of progressive T2DN by significantly suppressing the glomerular matrix expansion and progressive renal fibrosis such as a marked accumulation of collagen I, IV, FN, and α-SMA+ myofibroblasts (Figure 5A-F). Western blot and real-time PCR analysis further confirmed these notions that SIS3 treatment significantly inhibited progressive renal fibrosis by suppressing collagen I, FN, and α-SMA mRNA and protein expression on db/db mice despite α-SMA protein showed only a trend decrease (Figure 5G-M).

**Figure 6:**
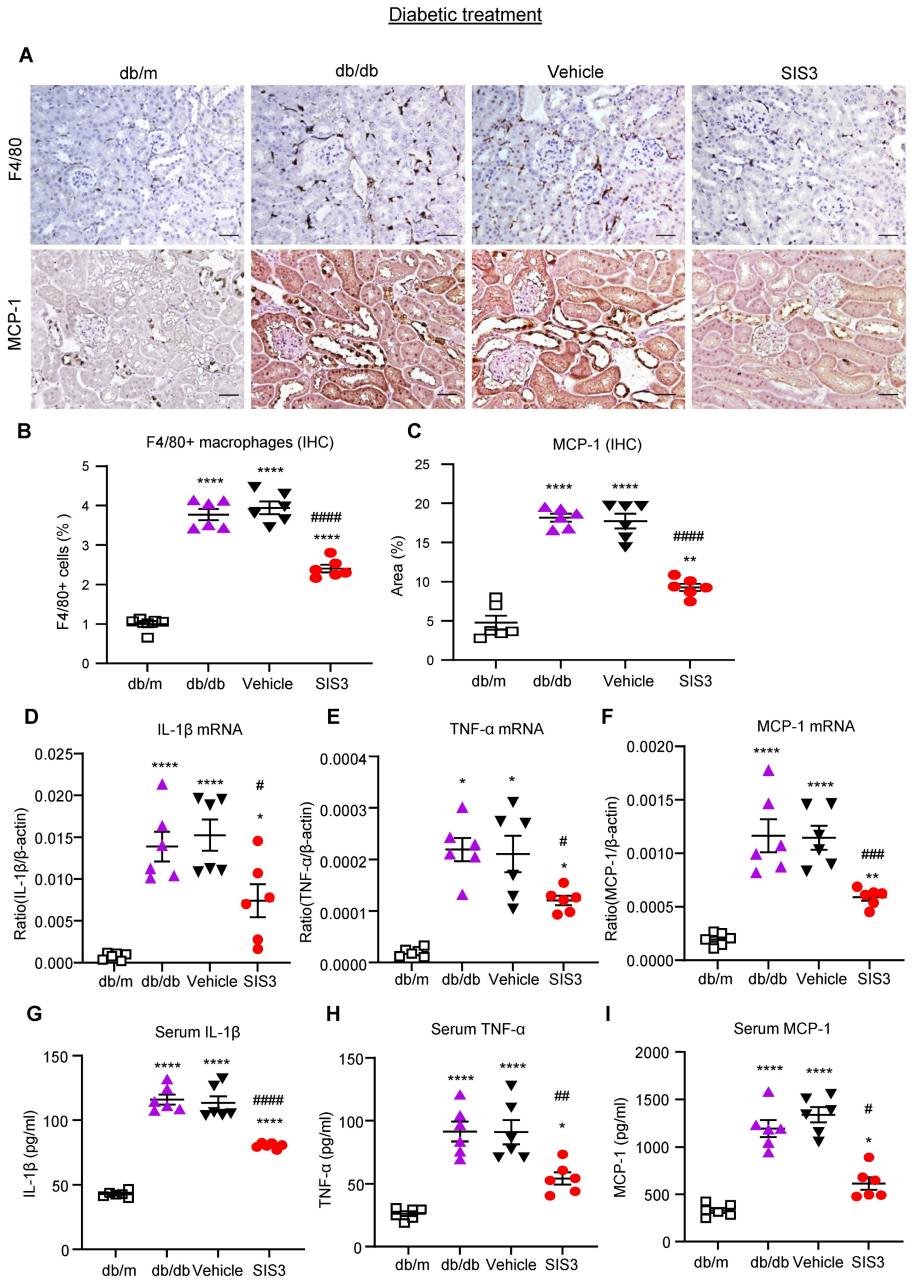
** Treatment of db/db mice with SIS3 from the age of 8 weeks to 16 weeks significantly inhibits renal inflammation in db/db mice. (A-C)** Immunostaining and quantitative analysis of F4/80+ macrophages and expression of MCP-1 in the kidney of db/m and db/db mice at the age of 16 weeks of age. **(D-F)** Real-time PCR analysis detects IL-1β, TNF-α, and MCP-1 mRNA expression in the kidney of db/m and db/db mice at 16 weeks.** (G-I)** ELISA analysis detects serum levels of IL-1β, TNF-α and MCP-1 in db/m and db/db mice at 16 weeks. Data represents the mean ± SEM for at least 6 mice per group. ^*^P<0.05, ^**^P<0.01, ^****^P<0.0001 versus normal db/m mice. ^#^*P*<0.05,^ ##^*P*<0.01, ^###^*P*<0.001, ^####^*P*<0.0001 compared with the control-treated db/db mice (vehicle). SIS3 dosages used: SIS3=SIS3 2.5 mg/kg.bw. Scale bar, 50μm.

We also examined the therapeutic effect of SIS3 on renal inflammation in db/db mice by using immunohistochemistry, real-time PCR, and ELISA. Similar to the findings in renal fibrosis, the 8-week-treatment with SIS3 in established db/db mice from 8 to 16 weeks significantly inhibited renal inflammation by suppressing F4/80+ macrophage infiltration and a marked upregulation of MCP-1, IL-1β, and TNF-α both locally and systematically (Figure 6, Supplementary Figure S6). Taken together, unlike the prediabetic treatment, treatment with SIS3 in the established diabetes did not effectively inhibit T2D but did significantly suppress progressive T2DN.

### Rebalancing Smad3/7 signaling and inhibition of Smad3-dependent lncRNA Erbb4-IR are mechanisms through which SIS3 inhibits renal fibrosis in db/db mice

**Figure 7:**
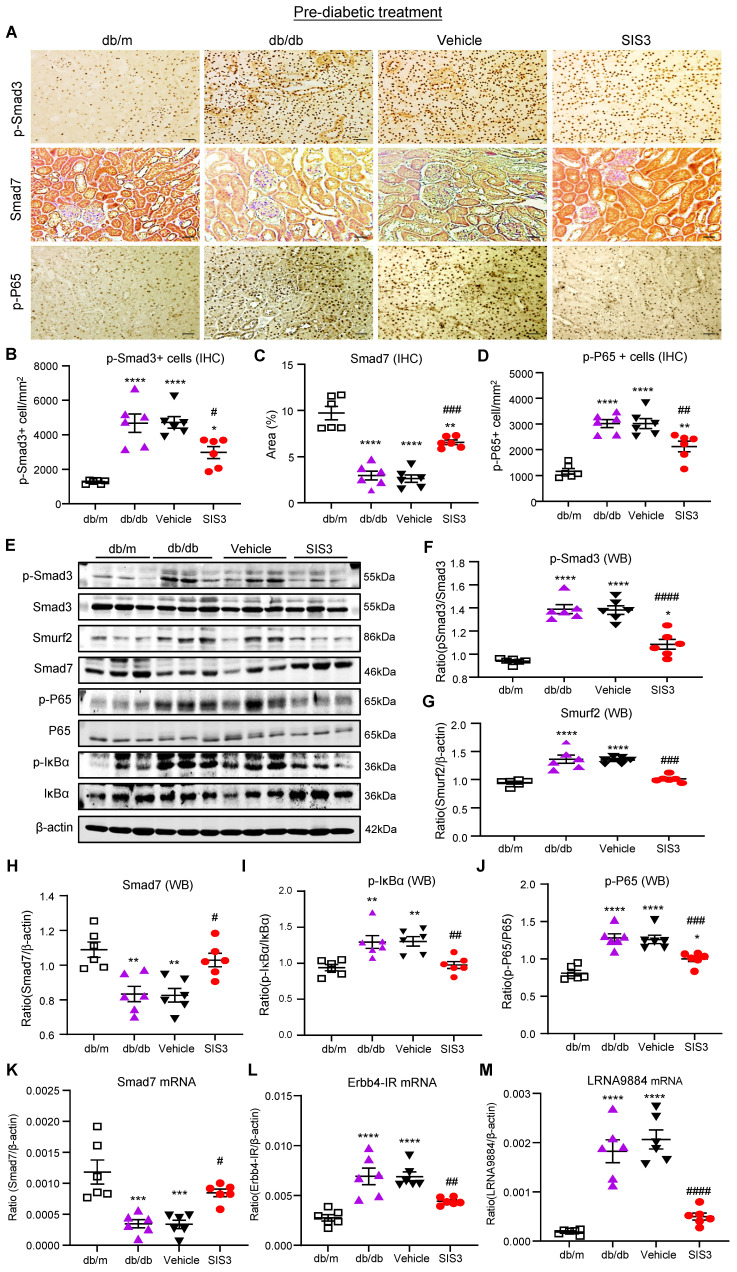
** Prediabetic treatment of db/db mice with SIS3 from the age of 4 weeks to 12 weeks restores the balance of Smad3/7 signaling, inactivates NF-kB/p65, and inhibits lncRNAs Erbb4-IR and LRNA9884 in the diabetic kidney of db/db mice. (A-D)** Immunostaining and semi-quantitative analysis for phospho-Smad3 (p-Smad3), Smad7, and p-p65 NF-kB subunit in the kidney of db/m and db/db mice at 12 weeks.** (E-J)** Western blots and quantitative analysis for p-Smad3, Smurf2, Smad7, p-p65, and p-IkBα in the kidney of db/m and db/db mice at 12 weeks. **(K-M)** Real-time PCR analysis detects Smad7 mRNA, lncRNA Erbb4-IR, and LRNA9884 expression in the kidney of db/m and db/db mice at 12 weeks. Data represents the mean ± SEM for at least 6 mice per group. ^*^P<0.05, ^**^P<0.01, ^***^P<0.001, ^****^P<0.0001 versus normal db/m mice. ^#^*P*<0.05, ^##^*P*<0.01, ^###^*P*<0.001, ^####^*P*<0.0001 compared with the control-treated db/db mice (vehicle). SIS3 dosages used: SIS3 =SIS3 2.5 mg/kg.bw. Scale bar, 50μm.

**Figure 8:**
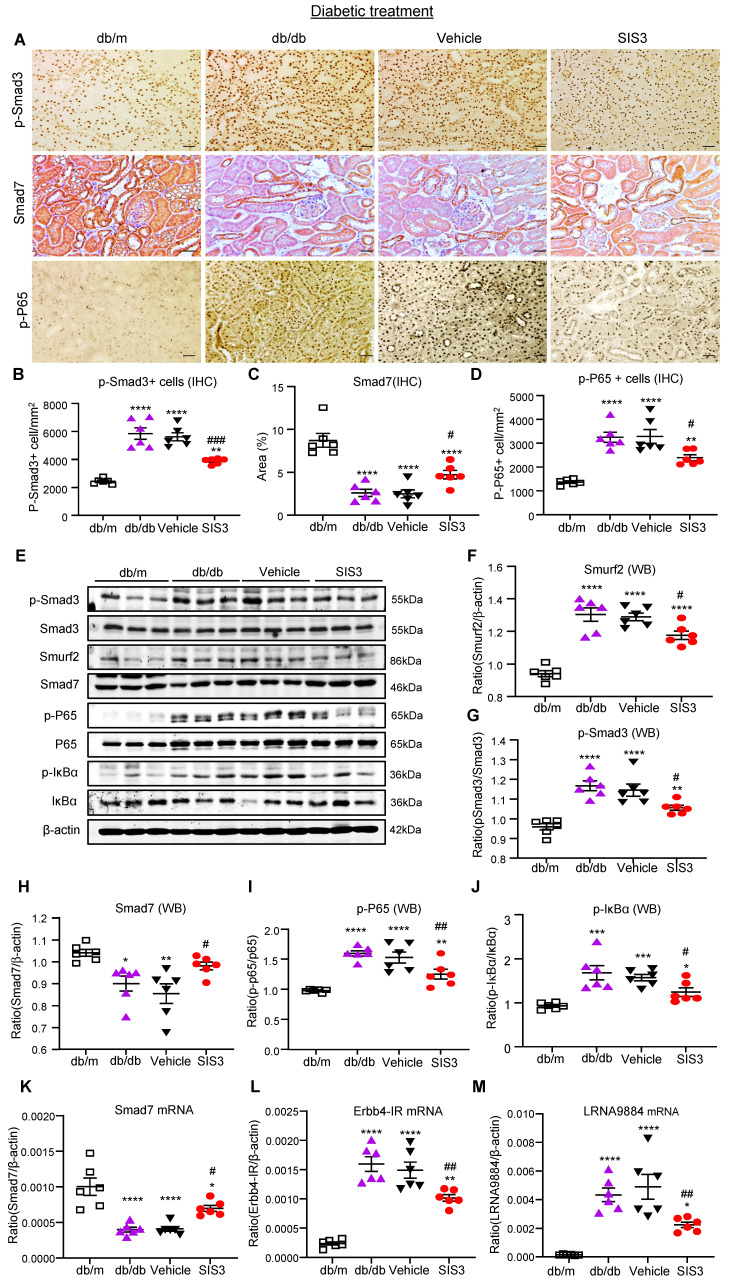
** Treatment of db/db mice with SIS3 from the age of 8 weeks to 16 weeks restores the balance of Smad3/7 signaling, inactivates NF-kB/p65, and inhibits lncRNAs Erbb4-IR and LRNA9884 in the diabetic kidney of db/db mice. (A-D)** Immunostaining and semi-quantitative analysis for phospho-Smad3 (p-Smad3), Smad7, and p-p65 NF-kB subunit in the kidney of db/m and db/db mice at 16 weeks. **(E-J)** Western blots and quantitative analysis for Smurf2, Smad7, p-Smad3, p-p65, and p-IkBα in the kidney of db/m and db/db mice at 16 weeks. **(K-M)** Real-time PCR analysis detects Smad7 mRNA, lncRNA Erbb4-IR, and LRNA9884 expression in the kidney of db/m and db/db mice at 16 weeks. Data represents the mean ± SEM for at least 6 mice per group. ^*^P<0.05, ^**^P<0.01, ^***^P<0.001, ^****^P<0.0001 versus normal db/m mice. ^#^*P*<0.05, ^##^*P*<0.01, ^###^*P*<0.001 compared with the control-treated db/db mice (vehicle). SIS3 dosages used: SIS3 =SIS3 2.5 mg/kg.bw. Scale bar, 50μm.

We next investigated the molecular mechanisms through which blockade of Smad3 inhibits renal fibrosis in T2DN in both prediabetic and diabetic-treated db/db mice. We have previously shown that deletion of Smad3 from db/db mice protects against diabetic complications by preventing Smad7 from the SMAD specific E3 ubiquitin protein ligase 2 (Smurf2)-dependent ubiquitin degradation 21, 22. Results shown in this study also clearly demonstrated that treatment with SIS3 significantly inhibited phosphorylation of Smad3 and its nuclear translocation, thereby preventing Smurf2-mediated Smad7 degradation in both prediabetic and diabetic-treated mice (Figure 7A-C, E-H, K and Figure 8 A-C, E-H, K).

We have also previously reported that Smad3 mediates renal fibrosis in obstructive nephropathy and T2DN via a lncRNA Erbb4-IR-dependent mechanism 31, 32. We thus examined whether treatment with SIS3 inhibits renal fibrosis in T2DN via a Smad3-dependent Erbb4-IR mechanism. Results shown in Figure 7 (L) and Figure 8 (L) revealed that treatment with SIS3 in both prediabetic and diabetic db/db mice significantly suppressed the expression of Erbb4-IR. Thus, it is possible that treatment with SIS3 inhibits T2DN by blocking Smad3-mediated Erbb4-IR expression.

### SIS3 treatment inhibits T2DN by blocking NF-kB-driven and lncRNA 9884-mediated renal inflammation in both prediabetic and diabetic-treated db/db mice

We have also previously reported that Smad7 can inactivate NF-κB signaling by inducing IκBα expression, an inhibitor of NF-kB signaling, while inhibiting its degradation 33, 34. As SIS3 treatment markedly upregulated renal Smad7 in both prediabetic and diabetic-treated db/db mice (Figure 7A, C, E, H, K and Figure 8A, C, E, H, K), we then examined whether SIS3 inhibits renal inflammation in T2DN by blocking NF-kB-driven renal inflammation. Indeed, immunohistochemistry and western blot analysis revealed that NF-kB signaling was highly activated in the diabetic kidney of db/db mice as demonstrated by a marked phosphorylation of IkBa and p65 and the phospho-p65 nuclear translocation, which was blocked by SIS3 treatment in both prediabetic and diabetic db/db mice (Figured 7A, D, E, I, J and Figure 8 A, D, E, I, J).

In addition, LRNA 9884 is also identified as a Smad3-dependent lncRNA associated with renal inflammation in T2DN via the MCP-1-dependent mechanism 35. We thus examined LRNA9884 expression in the diabetic kidneys of db/db mice treated with or without SIS3. As shown in Figure 7(M) and Figure 8 (M), LRNA9884 was largely upregulated in the kidney of db/db mice, which was almost completely inhibited by SIS3 treatment in both prediabetic and diabetic db/db mice, revealing that blockade of LRNA9884 expression may be another mechanism associated with the inhibition of renal inflammation in SIS3-treated db/db mice.

### Prediabetic treatment with SIS3 protects against T2D in db/db mice by promoting islet β cell development via the Pax6-dependent mechanism

**Figure 9:**
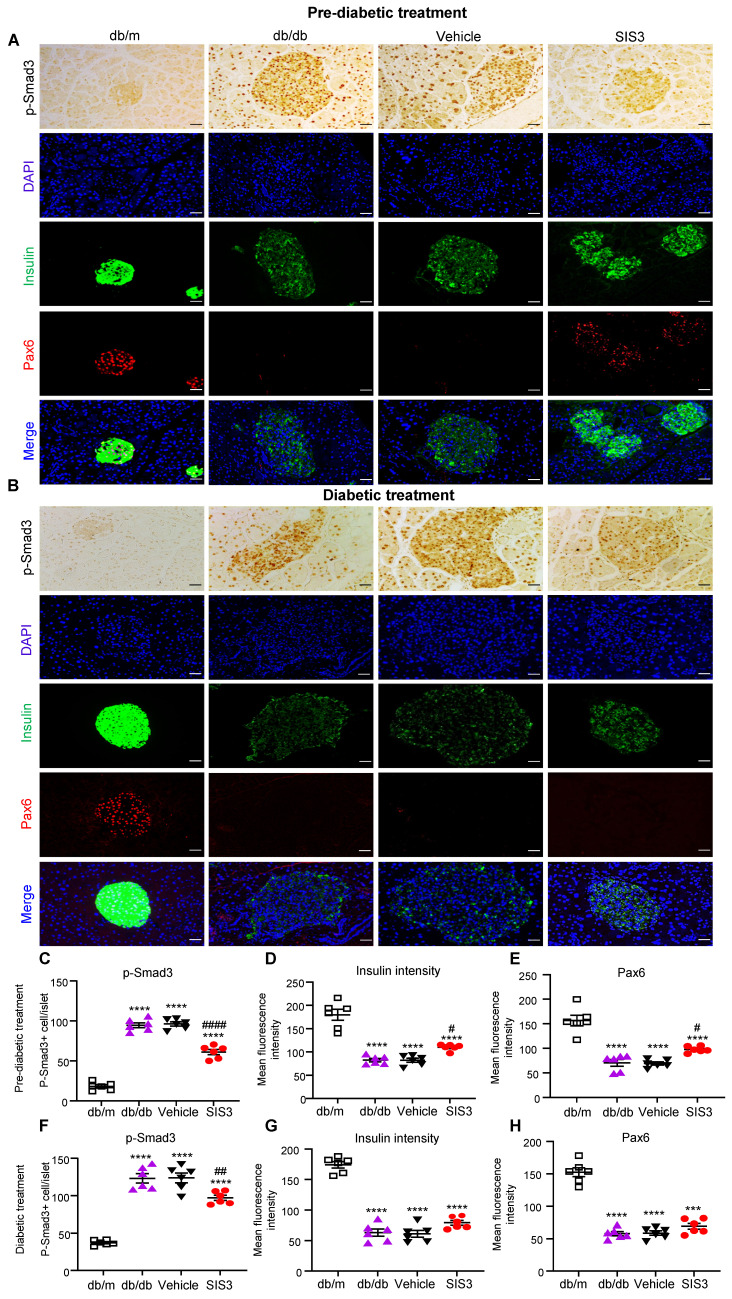
** Differential effect of prediabetic versus late diabetic treatment with SIS3 on islet Pax6 expression and insulin-producing b cell function in the pancreas islets of db/db mice. (A)** Immunohistochemistry and multiplex immunofluorescence for detecting p-Smad3, Pax6 and insulin-producing b cells in the pancreas islets of db/m and db/db mice that receive SIS3 (2.5 mg/kg/day) treatment from the prediabetic stage at the age of 4 weeks to 12 weeks. **(B)** Immunohistochemistry and multiplex immunofluorescence for detecting p-Smad3, Pax6 and insulin-producing b cells in the pancreas islets of db/m and db/db mice that receive SIS3 (2.5 mg/kg/day) treatment from the age of 8 weeks to 16 weeks.** (C)** Quantitative analysis of p-Smad3, insulin-producing islet cells, and Pax6+ islet cells from the prediabetic-treated db/db mice. **(D)** Quantitative analysis of p-Smad3, insulin-producing islet cells, and Pax6+ islet cells from the late SIS3-treated db/db mice. Data represents the mean ± SEM for at least 6 mice per group. ^***^P<0.001, ^****^P<0.0001 versus normal db/m mice. ^#^*P*<0.05, ^##^*P*<0.01, ^####^*P*<0.0001 compared with the control-treated db/db mice (vehicle). SIS3 dosages used: SIS3 =SIS3 2.5 mg/kg.bw. Scale bar, 50μm.

We have previously reported that genetic deletion of Smad3 from db/db mice protects against the development of T2D by restoring Pax6-dependent β cell proliferation and functions 20. In the present study, we revealed a significant loss of Pax6 and insulin-producing islet cells in untreated or vehicle-treated db/db mice at the age of 12 and 16 weeks. Unexpectedly, the early prediabetic treatment with SIS3 largely blocked islet Smad3 signaling and therefore protected against the loss of islet Pax6 expression and islet β cells injury as demonstrated by significantly increasing insulin-producing islet population (Figure 9A, C-E). In contrast, the late SIS3 treatment in the established db/db mice showed less inhibitory effect on islet Smad3 activation, resulting in the loss of islet Pax6 and insulin-producing islet β cells (Figure 9B, F-H). These findings revealed that the protection of islet β cells from the diabetic degenerative injury by preventing the loss of islet Pax6 may be a mechanism by which the early prediabetic but not the late diabetic treatment with SIS3 protected against the development of T2D in db/db mice.

## Discussion

In this study, we discovered that Smad3 is a therapeutic target for T2D and T2DN and Smad3-targeted therapy with a Smad3 inhibitor SIS3 can protect db/db mice from the development of T2D and T2DN. This is consistent with a previous finding that inhibition of Smad3 can block endothelial-mesenchymal transition and delays the early development of streptozotocin-induced T1DN 36. Interestingly, inhibition of Smad3 signaling by treating the prediabetic db/db mice from the age of 4 weeks to 12 weeks could protect against the development of both T2D and T2DN. However, it failed to prevent the development of T2D when db/db mice received the late treatment with SIS3 from the age of 8-16 weeks, although it also attenuated T2DN. These findings were consistent with our recent findings that the early prediabetic but not the diabetic treatment on db/db mice with the combined Smad7 agonist Asiatic acid and Smad3 inhibitor Naringenin (AANG) can effectively prevent T2D and T2DN 37. These findings are highly significant and meaningful clinically because there is an increasing prevalence of prediabetes and the young-onset of T2D and T2DN in adolescents and young adults 26. It is now well recognized that the young-onset of T2D is an urgent challenge clinically because patients with the young-onset T2D show more progressive diabetic complications with high socioeconomic burden 26, 27. Results from this suggest that the early treatment in the prediabetic stage may be able to effectively prevent the development of T2D and T2DN, particularly for those individuals with high risk with the young-onset of T2D clinically.

Mechanically, we uncovered that treatment with SIS3 inhibited T2DN in db/db mice by inhibiting Smad3 while upregulating Smad7, thereby restoring the balance of Smad3/7 signaling and protecting the kidney from the development of progressive renal fibrosis in db/db mice. These findings were also consistent with our recent findings that genetic deletion of Smad3 from db/db mice or treatment of db/db mice with AANG can block Smad3 activation while upregulating Smad7, thereby preventing the development of renal and cardiac fibrosis 21, 22, 37. It is now well accepted that in the context of renal fibrosis, Smad3 is pathogenic while Smad7 is protective 6, 7. Thus, overexpression of renal Smad7 can inhibit T2DN 38. Once Smad3 is activated, it can induce overexpression of Smurf2 that targets Smad7 for degradation, thereby disrupting the balance between Smad3 and Smad7 signaling 6, 7. Thus, treatment with SIS3 resulted in rebalancing Smad3/Smad7 signaling by inhibiting Smad3 while upregulating Smad7. This may be a mechanism through which SIS3 treatment attenuated T2DN in both prediabetic and diabetic treatment. Furthermore, we also identified that blockade of lncRNA Erbb4-IR could be another mechanism through which SIS3 treatment inhibited renal fibrosis in T2DN. It is known that Erbb4-IR is a Smad3-targeted gene that mediates renal fibrosis and silencing Erbb4-IR can inhibit T2DN and obstructive nephropathy 31, 32. Thus, inhibition of Erbb4-IR may be another mechanism by which treatment with SIS3 suppressed renal fibrosis in T2DN.

Inhibition of NF-kB-driven renal inflammation could be a mechanism by which treatment with SIS3 inhibited renal inflammation in T2DN. We previously reported that Smad7 not only functions to inhibit TGF-β/Smad signaling but also blocks NF-kB activation by inducing IkBα while inhibiting its degradation 33, 34. In the diabetic kidney, Smad7 is lost, which is mediated by the Smad3-Smurf2-dependent ubiquitin degradation mechanism, whereas overexpression of renal Smad7 inhibits T2DN by suppressing NF-kB-driven renal inflammation 38. In the present study, treatment with SIS3 inhibited Smad3-Smurf2-mediated Smad7 degradation pathway, thereby preventing renal Smad7 from degradation and blocking NF-kB-dependent renal inflammation. In addition, Smad3 can also induce LNRA9884 to cause renal inflammation in T2DN 35. Thus, inhibition of LRNA9884 may be another mechanism by which SIS3 treatment blocked renal inflammation in T2DN.

Importantly, we also found that the protection of islet β cells from diabetic injury by preventing the loss of islet Pax 6 may be a mechanism whereby prediabetic treatment with SIS3 inhibited T2D. Indeed, treatment with SIS3 in prediabetic db/db mice, but not in the established diabetic db/db mice, was able to inhibit the T2D phenotype including the elevated blood glucose and HbA1c and the development of insulin resistance and glucose tolerance. This was associated with inhibition of islet Smad3 activation while increasing Pax 6 expression and insulin production. This finding was consistent with our previous finding that db/db mice lacking Smad3 are protected from the development of T2D by preventing Smad3-mediated loss of islet Pax 6, thereby increasing islet β cell proliferation and functions 20. It is well accepted that the onset and progression of T2D are associated with the insufficient insulin secretion caused by pancreatic β-cell dysfunction and insulin resistance in target organs 3, 4. Pax6 is a key regulator of α- and β-cell differentiation, and is essential for insulin biosynthesis, insulin secretion and glucose homeostasis 39-41. The MH1 domain of Smad3 can interact with Pax 6 and represses Pax 6 expression 42. Therefore, TGF-β/Smad3 signaling is crucial in regulating β cell dysfunction by suppressing Pax 6 expression 43-45. It is possible that inhibition of Smad3-mediated loss of islet Pax 6 may be a mechanism through which the prediabetic treatment with SIS3 improved the T2D phenotype in db/db mice. However, this protective effect was lost when the late SIS3 treatment was given to the established db/db mice. This may be associated with the ineffectiveness of the late SIS3 treatment on overreactive Smad3 signaling. This differential effect between prediabetic and diabetic SIS3 treatment on islet Pax 6 expression may well explain the notion that the prediabetic but not the diabetic SIS3 treatment inhibited T2D.

Another interesting finding in this study was that although the late SIS3 treatment in the established db/db mice failed to protect against the development of T2D, it did produce an inhibitory effect on T2DN. Although the mechanisms related to these differential effects are largely unclear, it may be associated with the difference in the SIS3 accessibility to the islet and the kidney. Because kidney is much rich in blood circulation when compared to the islet, it is possible that SIS3 is much more accessible to the kidney than the islet. This may explain the finding that SIS3 treatment significantly blocked the intrarenal TGF-β/Smad3 signaling and T2DN with minimal effect on protection against Pax 6 loss and T2D as seen in this study in established db/db mice. Taken together, results from this study revealed that targeting Smad3 may be a novel therapy for T2D and T2DN. Importantly, findings from this study also indicate that T2D is preventable if early anti-Smad3 treatment was given, with more favorable in the prediabetic stage.

## Material and methods

### Animal model and SIS3 treatment

The db/db mice with mutation of Lepr (Leptin receptor) in C57BLKS background were purchased from the Laboratory Animal Services Centre of The Chinese University of Hong Kong (CUHK). All animal husbandry and animal experiments in this study were approved by the Animal Experimentation Ethics Committee of CUHK (Ref No.: 17-559 in DH/SHS/8/2/1 Pt.4) and were conducted in the Laboratory Animal Services Centre of CUHK and Animal Unit of Prince of Wales Hospital. All mice were bred and housed under specific pathogen free (SPF) conditions in a temperature-controlled room (25 °C) with a 12-h dark/light cycle setting.

To determine a safe and effective dose of SIS3 for treatment of db/db mice, groups of 6-8 male db/db and normal db/m mice were intraperitoneal (i.p.) injected with solvent (DMSO) diluted SIS3 (Selleck Chemicals, Houston, TX, USA; cat# S7959) at dosages of 1.25, 2.5, 5mg/kg.bw/day for 8 weeks from the age of 4 weeks to 12 weeks. Vehicle control mice followed the same protocol but received the solvent (0.9% NaCl supplementary with 0.1% DMSO and 5% Tween 80). In addition, a group of 6-8 male 4-week db/m mice and a group of 6-8 male 4-week db/db mice were used as normal and untreated disease controls.

After determining an optimal dose of SIS3 (2.5 mg/kg.bw/day) that effectively inhibited T2D without systemic toxicities (Figure 1 and Figure S1), we first treated pre-diabetic db/db mice by i.p. injection with SIS3 at dosage of 2.5 mg/kg.bw/day from the age of 4 weeks to 12 weeks. Then, we studied the therapeutic effect of SIS3 on established T2D in db/db mice by i.p. injection with SIS3 at dosage of 2.5 mg/kg.bw/day from the age of 8 weeks to 16 weeks. Control-treated db/db mice were given with the solvent, following the same experimental protocol as described above.

The therapeutic effect of SIS3 on the development of T2D and T2DN was determined by weekly body weight and fasting blood glucose, intraperitoneal glucose tolerance test (IPGTT), intraperitoneal insulin tolerance test (IPITT), serum levels of HA1c and creatinine, and microalbuminuria at the time of sacrifice after 8 weeks of treatment. All mice were sacrificed by i.p. injection of phenobarbital. Blood and tissues were collected for analysis as described below.

### Fasting blood glucose, body weight

After overnight (12 h) fasting, mice were examined for fasting blood glucose levels weekly by Accu-Chek glucose meter (Roche Diagnostics, Indianapolis, IN). For body weight measurements, mice were individually weighed weekly over the 8-week period.

### Glucose and Insulin Tolerance Tests

For glucose tolerance tests (IPGTT), mice were fasted overnight (12 hours) and given i.p. injection of glucose (1mg/g body weight). Tail tip blood glucose levels were determined at 0, 15, 30, 60 and 120 min after injection. For insulin tolerance tests (IPITT), mice were fasted for 5 h and given i.p. injection of insulin (1 U/kg body weight). Blood glucose levels were determined at 0, 15, 30, 60 and 120 min after injection as previously described 18, 20.

### Renal Function Measurement

24-hour urinary samples were collected from mice by housing individual mice in metabolic cages. Urinary microalbumin was measured by competitive ELISA according to the manufacturer's instructions (Exocell, Philadelphia, PA, USA, cat# 1011). Urinary creatinine was measured by an enzymatic kit (Stanbio Laboratories, TX, USA, cat# 0420-500) according to manufacturer's instructions. Urinary albumin excretion was expressed as total urinary albumin/creatinine ratio (UACR, μg/mg) as previously study 21. Serum creatinine was determined accordingly with enzymatic method (Stanbio Laboratories, TX, USA). All measurements were performed as previously described 15, 18, 21.

### Biochemical Measurements and ELISA Analysis

Serum aspartate aminotransferase (AST), alanine aminotransferase (ALT) and lactate dehydrogenase (LDH) were determined by following the manufacturer's instructions (Nanjing Jiancheng Bioengineering Institute, Nanjing, China). Serum IL-1β, serum TNF-α and serum MCP-1 were determined according to the manufacturers' instructions (PeproTech Asia, Rehovot, Israe, cat# 900-K47, cat# 900-K54, cat# 900-K126). Fasting serum insulin was determined by following the manufacturer's instructions (ImmunoDiagnostics Limited, Hongkong, China, cat# 32270).

### Histology and Immunohistochemistry

Kidney tissues were fixed in methyl Carnoy's solution, embedded in paraffin, and sectioned at 3 μm. The pancreas was fixed in 4% paraformaldehyde, embedded in paraffin, and sectioned at 4 μm. The primary antibodies used for immunohistochemistry included those against phospho-NF-κB/p65 (ser276) (Abcam, Cambridge, MA, USA. cat# 97726), phospho-Smad3 (Cell Signaling Technology, Berkeley, CA. cat# 9520), Smad7 (Abcam, cat# ab244424), collagen I and collagen IV (Southern Biotech, Birmingham, AL), fibronectin (FN) (Abcam, cat# ab2413), F4/80 (AbD Serotec, San Diego, CA. cat# MCA497G), TNF-α (Santa Cruz Biotechnology, Santa Cruz, CA. cat# sc-1351), IL-1β (Abcam, cat# 205924) and MCP-1 (Santa Cruz Biotechnology, cat# sc-1785). The Periodic Acid-Schiff (PAS) staining and immunohistochemical staining are as described previously 15, 18, 20-22, 24. The nuclei were counterstained with haematoxylin. The positive cells were counted under the high-power field (x20) of microscope in 10 random areas of kidney tissues and expressed as positive cells per square millimeter. The percentage of positive staining areas such as collagen matrix was quantified using Image J software (v1.48, NIH, USA) in 10 consecutive fields.

### Multiplex immunofluorescence

To detect the co-localization between Pax6 and insulin, paraffin-embedded pancreas sections (4 μm) were treated with 3% H2O2 to block endogenous horseradish peroxidase and then incubated with rabbit polyclonal antibody against Pax6 (Abcam, cat# ab195045) for overnight, followed by antibody against insulin (Abcam, cat# ab181547). Sections were incubated with EnVision+ System-HRP Labelled Polymer (Dako, Santa Clara, CA. cat# K4003) at room temperature for 1 hour, then the fluorescence were developed using Alex Fluor 568 Tyramide Reagent (Invitrogen, Carlsbad, CA, USA. cat# B40956) or Alexa Fluor 488 Tyramide Reagent (Invitrogen, cat# B40953) according to the manufacturer's protocol. The nuclei were counterstained with Hoechst 33342 (Invitrogen, cat# H1399). All quantitation was performed with Image J software as previously described 20.

### Western blotting analysis

Total protein from renal cortical tissues were extracted by RIPA lysis buffe, and western blotting analysis was performed as previously described 15-18, 20-22, 24. Antibodies used in this study included those against phospho-NF-κB/p65 (ser276) (Cell Signaling Technology, Berkeley, CA. cat# 3033), phospho-IκBα (ser32) (Cell Signaling Technology, cat# 2859), IκBα (Cell Signaling Technology, cat# 9242), phospho-Smad3 (Abcam, cat# ab52903), Smad3 (Invitrogen, cat# PA1-38613), Smad7 (Santa Cruz Biotechnology, cat# sc-9183), Smurf2 (Santa Cruz Biotechnology, cat# sc-2511), NF-κB/p65 (Cell Signaling Technology, cat# 8242), β-actin (Santa Cruz Biotechnology, cat# sc-47778), collagen I (Southern Biotech), FN (Abcam, cat# ab2413). After being incubated with the primary antibody at 4 °C overnight, the membrane was stained with the LI-COR IRDye 800-labeled secondary antibodies (1:3000, Rockland Immunochemicals, Gilbertsville PA, USA) for 1h. The signals were detected with Odyssey Infrared Imaging System (Li-COR Biosciences, Lincoln, NA, USA) and quantitated with Image J software (v1.48, NIH, USA). The intensity of the protein band was normalized against β-actin or total proteins as stated in the studies and expressed as the mean ± SEM.

### RNA Extraction and Quantitative real-time PCR

RNA was extracted from renal cortical tissues by using Trizol (Invitrogen, CA; cat# 15596026), according to the manufacturer's instructions, and real-time PCR was performed using Bio-Rad IQ SYBR Green Supermix with Option 2 (Bio-Rad, Hercules, CA, USA). The Sequence of RNA Primers used such as Smad7, collagen I, FN, α-SMA, MCP-1, IL-1β, TNF-α, Erbb4-IR, LRNA9884, β-actin and TGF-β1 were described previously 15-18, 20-22, 24. The relative level of the detected gene was normalized with the internal control β-actin by the delta-delta Ct method and expressed as mean ± SEM.

### Statistical analysis

All the data are expressed as mean ± SEM. Statistical analysis was performed by one-way analysis of variance (ANOVA), followed by Newman-Keuls multiple comparison using Prism 7.0 (GraphPad Software, San Diego, CA). Statistical analysis among multiple groups were performed by two-way ANOVA, followed by a Newman-Keuls multiple comparisons test using Prism 7.0 (GraphPad Software, San Diego, CA). *p*-values below 0.05 were considered significant.

## Supplementary Material

Supplementary figures.

## Abbreviations

ANOVA: analysis of variance
ALT: alanine aminotransferase
AST: aspartate aminotransferase
AUC: area under curve
CKD: chronic kidney disease
Col-I: collagen I
Col-IV: collagen IV
DN: diabetic nephropathy
HbA1c: Hemoglobin A1C
IHC: immunohistochemistry
IL-1β: interleukin-1β
I.P.: intraperitoneal
IPGTT: intraperitoneal glucose tolerance test
IPITT: intraperitoneal insulin tolerance test
Kg. bw: kg body weight
KO: knockout
LncRNA: long noncoding RNA
Lepr: leptin receptor
MCP-1: monocyte chemoattractant protein-1
PAS: periodic acid-Schiff
PCR: polymerase chain reaction
p-Smad3: phospho-Smad3
p-IκBα: phospho-IkBα
p-NF-kB/65: phospho-NF-kB/65
SEM: standard error of the mean
STZ: streptozotocin
SIS3: specific inhibitor of Smad3
Smurf2: SMAD specific E3 ubiquitin protein ligase 2
T1DN: type 1 diabetic nephropathy
T2DN: type 2 diabetic nephropathy
T2D: type 2 diabetes
TGF-β: transforming growth factor-β
TNF-α: tumor necrosis factor-α
UUO: unilateral ureteral obstruction
UACR: Urine albumin/creatinine ratio
WB: Western blotting
WT: wild-type
