# Supplementary material for: Treatment for type 2 diabetes and diabetic nephropathy by targeting Smad3 signaling
Source: Int J Biol Sci. 2024 Jan 1;20(1):200–17. doi: 10.7150/ijbs.87820 (PMC10750285; doi:10.7150/ijbs.87820)
Supplement: Supplementary file 1 — Supplementary figures. [file ijbsv20p0200s1.pdf]

Pre-diabetic treatment

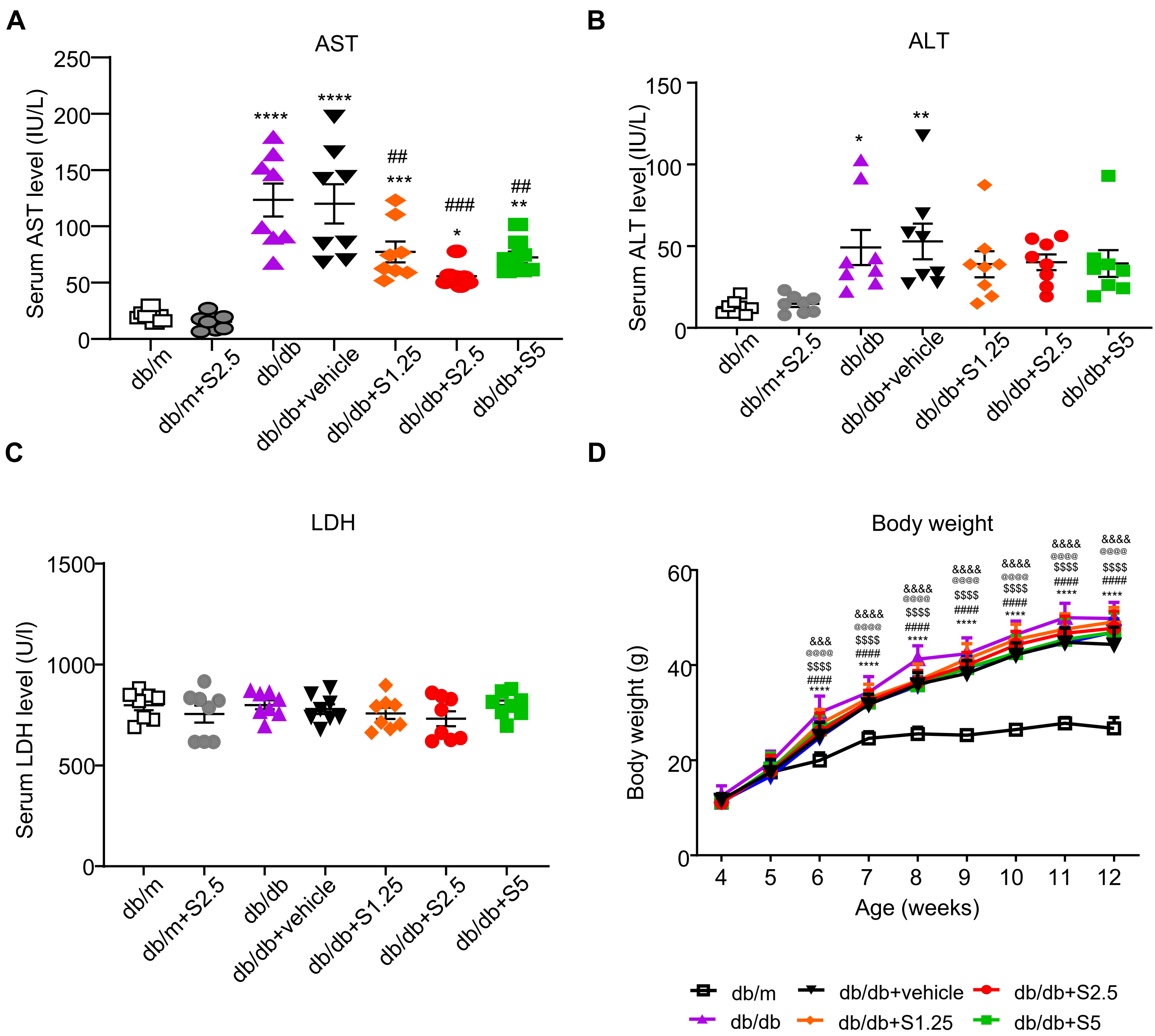

Supplementary Figure S1. Prediabetic treatment of db/db mice with SIS3 from the age of 4 weeks to 12 weeks in a dose-dependent manner shows no systemic toxicity in db/db mice. (A-C) The serum levels of AST, ALT, and LDH in db/m and db/db mice received 8 weeks of SIS3 treatment (weeks 4-12); $^{*}P$<0.05, $^{**}P$<0.01, $^{***}P$<0.001, $^{****}P$<0.0001 versus normal db/m mice; $^{\#\#}P$<0.01, $^{\#\#\#}P$<0.001 compared with the control-treated db/db mice (vehicle). (D) Effect of SIS3 treatment on body weight. $^{\#\#\#\#}P$<0.0001: S1.25-treated db/db mice versus normal db/m mice; $^{****}P$<0.0001: S2.5-treated db/db mice versus normal db/m mice; $^{@@@@}P$<0.0001 S5-treated db/db mice versus normal db/m mice; $^{\&\&\&}P$<0.001, $^{\&\&\&\&}P$<0.0001: vehicle-treated db/db mice versus normal db/m mice; $^{\$\$\$\$}P$<0.0001 untreated db/db mice versus normal db/m mice. Data represents the mean ± SEM for at least 6 mice per group. SIS3 dosages used: S1.25=SIS3 1.25 mg/kg.bw, S2.5=SIS3 2.5 mg/kg.bw, S5=SIS3 5 mg/kg.bw.

Diabetic treatment

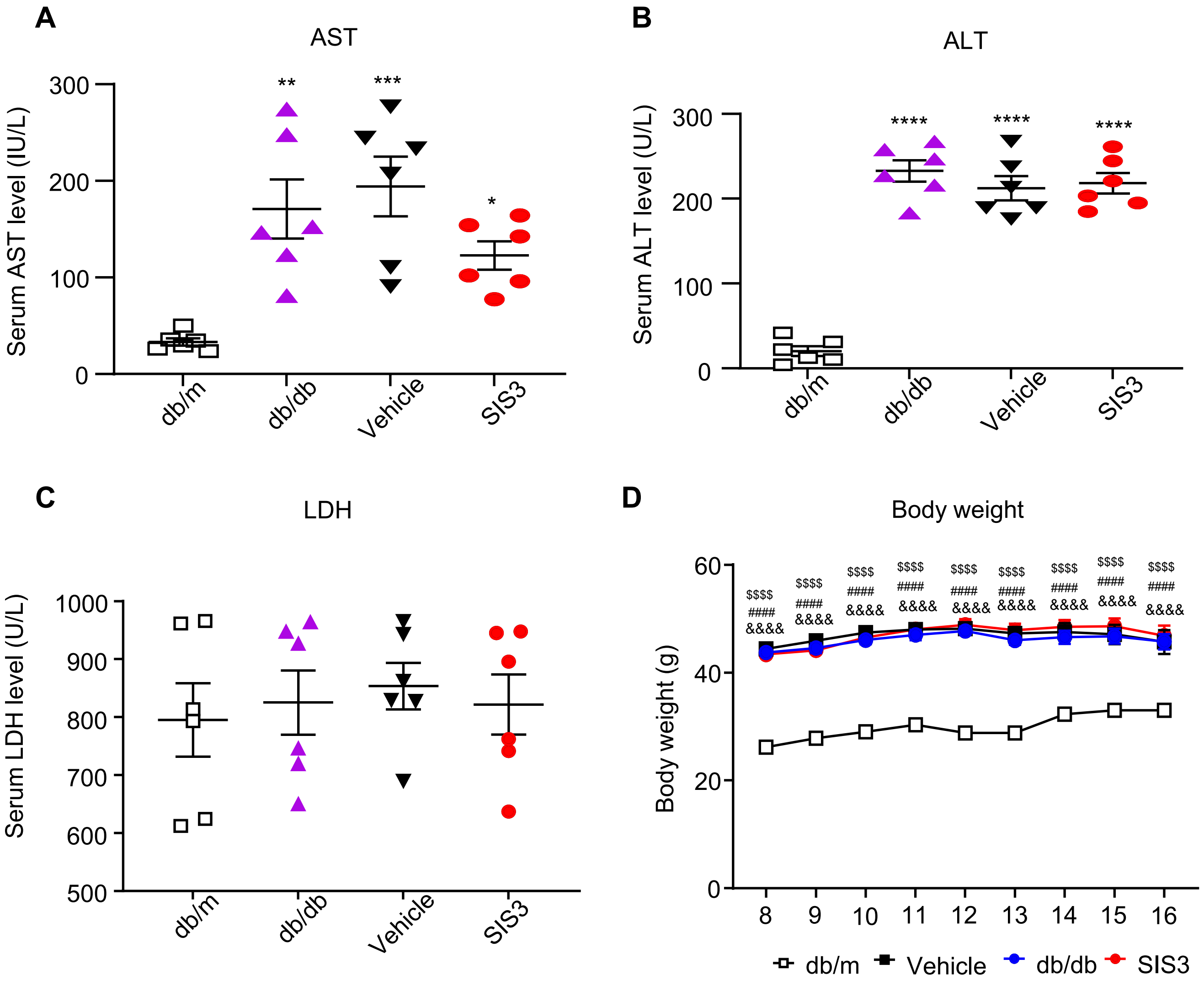

Supplementary Figure S2. Treatment of db/db mice aged 8 to 16 weeks with an optimal dose of 2.5 mg/kg SIS3 shows no systemic toxicity in db/db mice. (A-C) Serum ALT, AST and LDH levels at 16 weeks. $^{*}P<0.05$, $^{**}P<0.01$, $^{***}P<0.001$, $^{****}P<0.0001$ versus normal db/m mice. (D) Body weight at 16 weeks after SIS3 treatment (weeks 8-16). $^{\&\&\&\&}P<0.0001$: SIS3-treated db/db mice versus normal db/m mice. $^{\#\#\#\#}P<0.0001$: vehicle-treated db/db mice versus normal db/m mice. $^{\$\$\$\$}P<0.0001$: untreated db/db mice versus normal db/m mice. Data represents the mean ± SEM for at least 6 mice per group. SIS3 dosages used: SIS3=SIS3 2.5 mg/kg.bw.

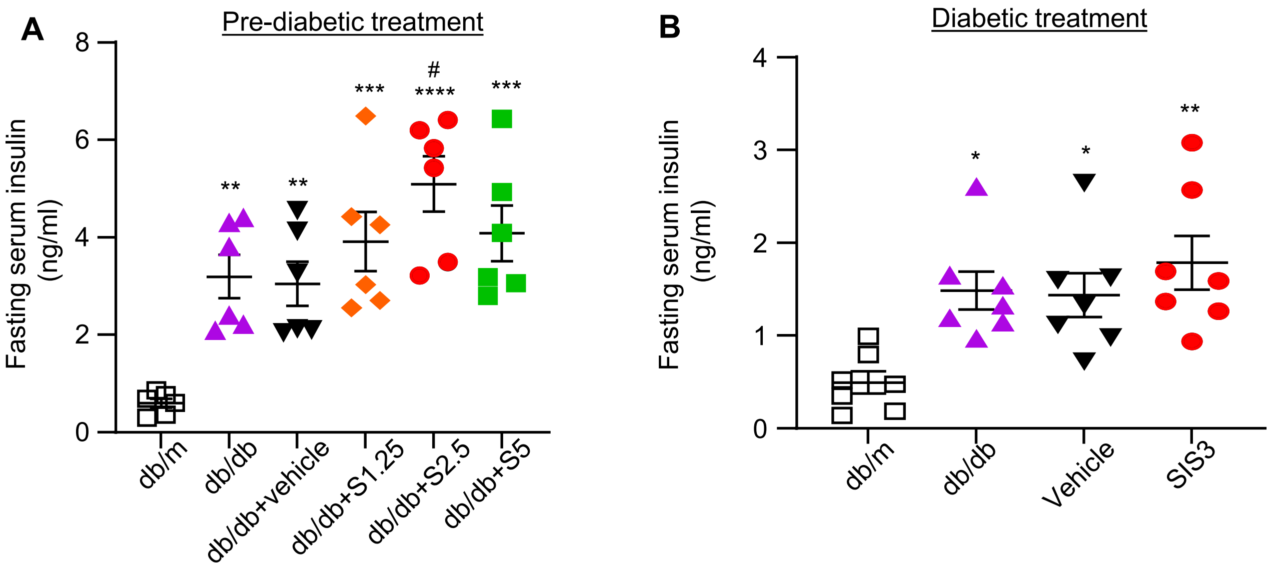

Supplementary Figure S3. Effect of SIS3 treatment on fasting serum insulin levels in prediabetic and diabetic db/db mice. (A) Fasting serum insulin at 12 weeks after SIS3 treatment (weeks 4-12). (B) Fasting serum insulin at 16 weeks after SIS3 treatment (weeks 8-16). Data represents the mean ± SEM for at least 6 mice per group. $^{*}P$<0.05, $^{**}P$<0.01, $^{***}P$<0.001, $^{****}P$<0.0001 versus normal db/m mice. $^{\#}P$<0.05 compared with the control-treated db/db mice (vehicle). SIS3 dosages used: S1.25=SIS3 1.25 mg/kg.bw, SIS3 or S2.5=SIS3 2.5 mg/kg.bw, S5=SIS3 5 mg/kg.bw.

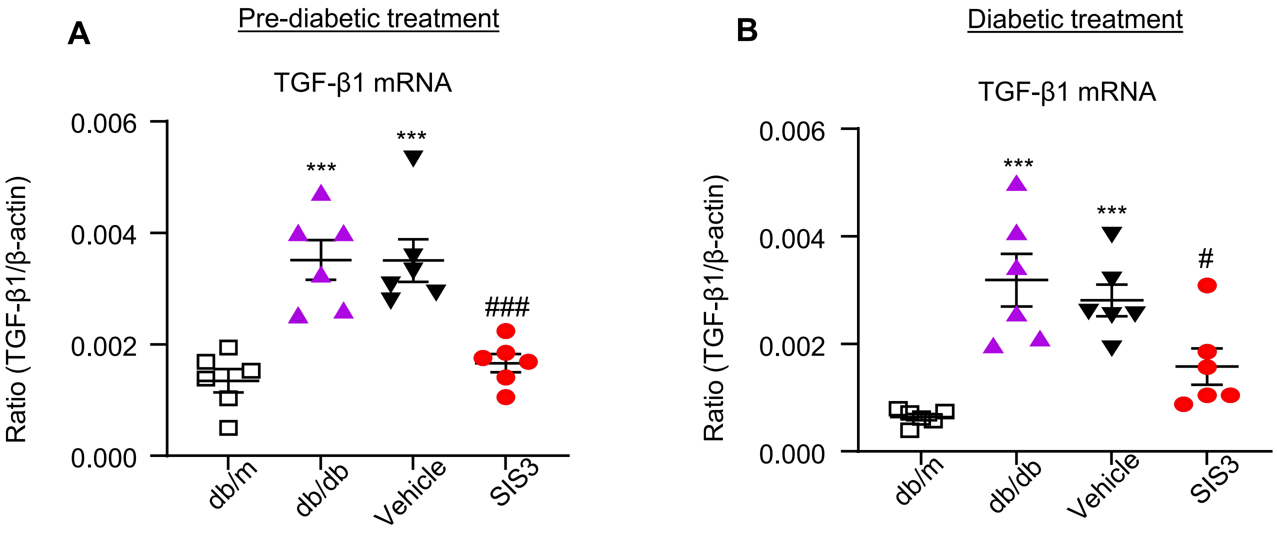

Supplementary Figure S4. Effect of SIS3 treatment on expression of TGF-β1 in renal tissues in prediabetic and diabetic db/db mice. (A) Real-time PCR analysis of TGF-β1 in renal tissues in db/db mice at 12 weeks after SIS3 treatment (weeks 4-12). (B) Real-time PCR analysis of TGF-β1 in renal tissues in db/db mice at 16 weeks after SIS3 treatment (weeks 8-16). Data represents the mean ± SEM for at least 6 mice per group. $^{***}P<0.001$ versus normal db/m mice. $^{\#}P<0.05$, $^{\#\#\#}P<0.001$ compared with the control-treated db/db mice (vehicle). SIS3 dosages used: SIS3 =SIS3 2.5 mg/kg.bw.

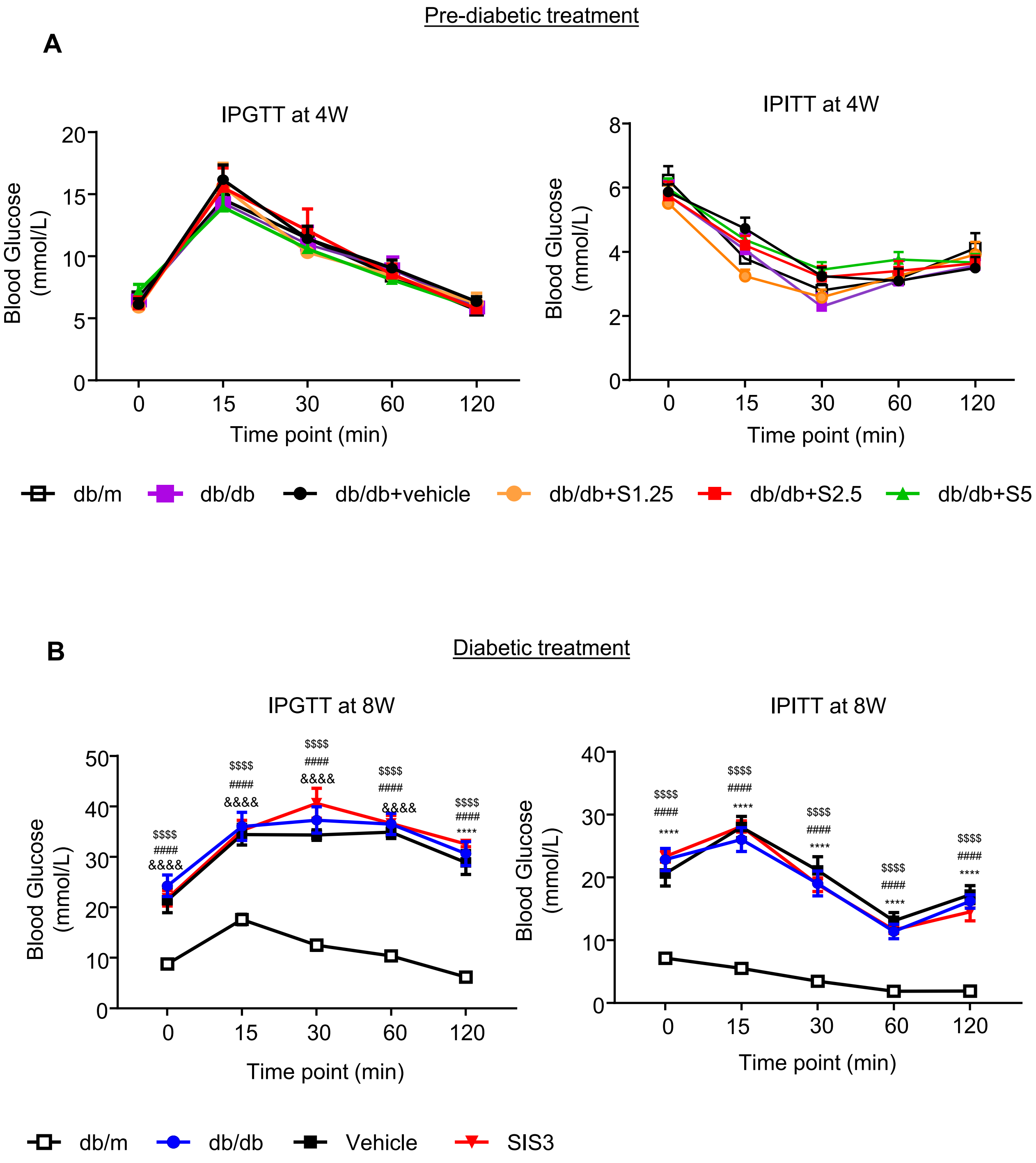

Supplementary Figure S5. Blood glucose levels during intraperitoneal glucose tolerance test (IPGTT) and intraperitoneal insulin tolerance test (IPITT) at 4 weeks or 8 weeks db/db mice. (A) IPGTT and IPITT in db/db mice at 4 weeks. $^{****}P<0.0001$ versus normal db/m mice. (B) IPGTT and IPITT in db/db mice at 8 weeks. $^{****}P<0.0001$: SIS3-treated db/db mice versus normal db/m mice; $^{\#\#\#\#}P<0.0001$: vehicle-treated db/db mice versus normal db/m mice; $^{\$\$\$\$}P<0.0001$: control-treated db/db mice versus normal db/m mice. Data represents the mean ± SEM for at least 6 mice per group. SIS3 dosages used: S1.25=SIS3 1.25 mg/kg.bw, SIS3 or S2.5=SIS3 2.5 mg/kg.bw, S5=SIS3 5 mg/kg.bw.

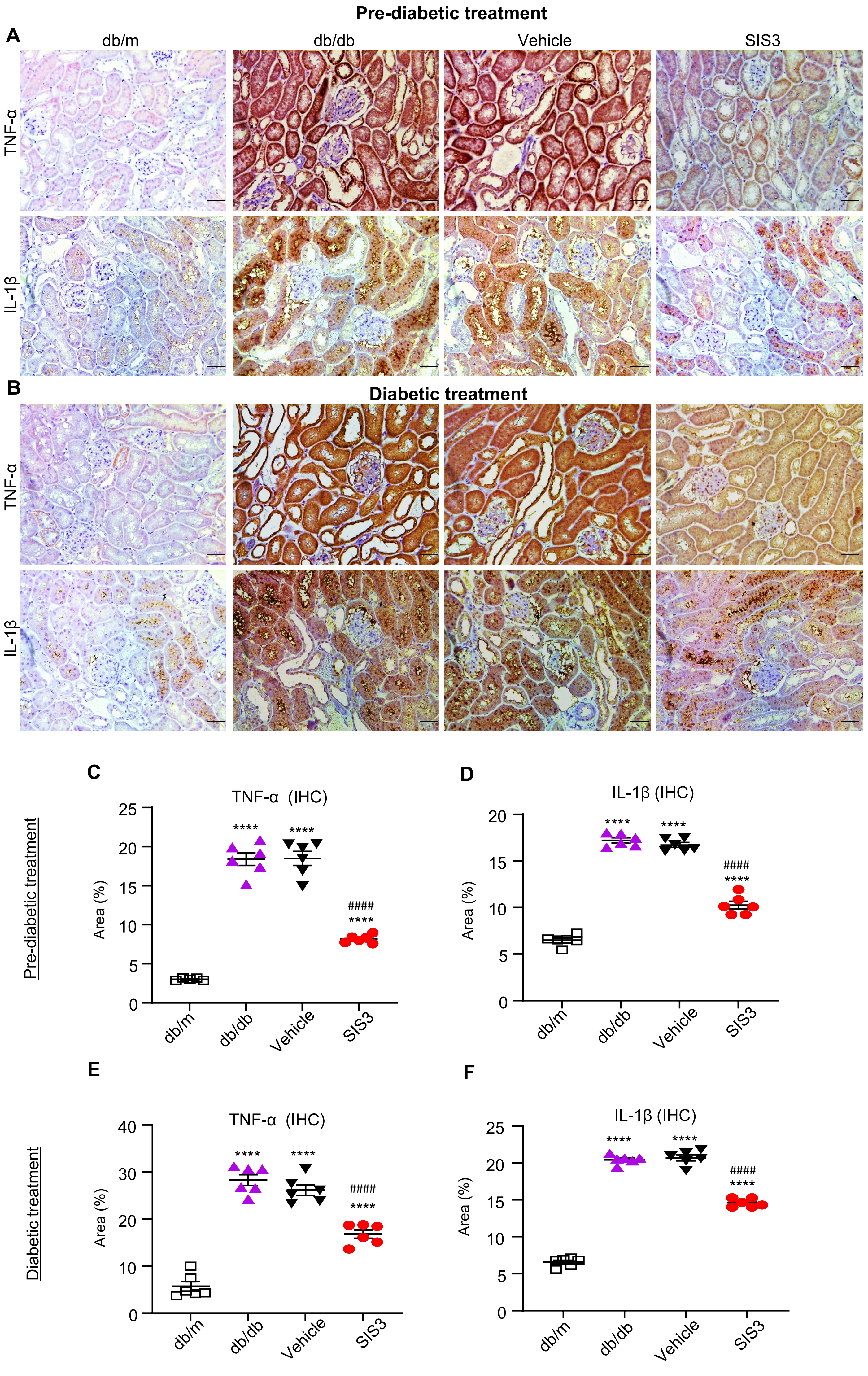

Supplementary Figure S6. Effect of prediabetic versus late diabetic treatment with SIS3 on kidney IL-1β and TNF-α expression in db/db mice. (A, C-D) Immunostaining and semi-quantitative analysis for IL-1β and TNF-α in the kidney of db/m and db/db mice at 12 weeks. (B, E-F) Immunostaining and semi-quantitative analysis for IL-1β and TNF-α in the kidney of db/m and db/db mice at 16 weeks. $^{****}P<0.0001$ versus normal db/m mice. $^{\#\#\#\#}P<0.0001$ compared with the control-treated db/db mice (vehicle). SIS3 dosages used: SIS3 =SIS3 2.5 mg/kg.bw. Data represents the mean ± SEM for at least 5 mice per group. Scale bar, 50μm.
